# The Triumph of the Voluntary Principle
*An address given at the Annual Meeting of the League of Mercy at St. James's Palace, December 19.


**Published:** 1916-12-23

**Authors:** R. J. Campbell


### THE TRIUMPH OF THE VOLUNTARY PRINCIPLE.*

### By the Rev. R. J. Campbell, M.A.

We are living in sad times; it has been the case with all similar periods of human history, and for that reason we have produced wonderful examples of faith, fortitude, and self-sacrifice. Given a great need, a great demand upon our individual and communal moral reserves, somehow we generally manage to respond to it, and occasionally in a surprising degree. At any rate, a sufficient number of us do so to make it possible to say that the soul of the nation, or rather—may I say?—the soul of the ancient race to which we belong, has been quickened into new and noble forms of activity. This has been the case with our own country in the trying two and a-half years through which we have passed, and I think the fact will be generally conceded by observers of the signs of the times. We have had much to deplore, and much to be ashamed of, both in manners and methods, during the war; but I think it may fairly be affirmed at the same time that, on the whole, our people have risen to the occasion and have acquitted themselves right well. Personally, I would be prepared to go further and say that, despite the miseries and the sorrows of this anxious time, we have, thus far, morally speaking, been the gainers, and not the losers, by what we have suffered and by what has been required of us in the way of personal service, as well as in the giving of our best and dearest for the maintenance of our cause on the battlefield.

### Spiritual Bankruptcy.

Civilisation was not in a very hopeful state before the war; at least, with all deference to other opinions, that is mine. We had become so ossified in our practical materialism as to be quite unaware of our spiritual bankruptcy. We did not know what we were living for: the sole aim and object of large classes of our population seemed to be the gratification of mundane desire, the avoidance of responsibility, and to have as good a time as possible, at the smallest cost of inconvenience and self-denial. I think idealism was well-nigh dead, a spurious philanthropy had taken its place. The usual tendency was more and more to look to the State for the easing of the individual burden and the healing of our social ills. There was one marked difference between the increasing call for State action in England as compared with that which prevailed in Germany. Here, I think, the tendency was more and more to regard the individual as the beneficiary of the State; there it was to regard the individual as existing only in and for the State. In England we were busily insisting on rights without duties, and in Germany they were insisting on duties without rights. This is a not unimportant difference, and accounts for much that has taken place in the last two and a-half years.

### Its Soundness and Worth.

What, then, may we say we have learned in the interval with respect to this one question of the relation of the individual to the State? Well, surely, for one thing, to distrust the tendency to rely upon the State to do anything for us. The State of the future, if we have learned our lesson rightly, must never be allowed to supersede or absorb into itself individual responsibility and voluntary effort. In the case of Germany we see what that has inflicted upon the world. The German State is a moral monster, a sort of Juggernaut, a soulless entity, intent only upon its own aggrandisement, at whatever cost, in over-riding considerations of truth and honour and humanity. In fact, as we are constantly being reminded, these things, in the German official point of view, have no meaning at all as applied to the State. The State can do whatever it feels to be, at the moment, for its own practical advantage. In England, on the other hand, we have been pulled up sharply by some menacing facts. We have been terribly reminded of the limits of State effectiveness in the matter of ameliorating the individual lot. Into the question of State organisation for war purposes I will not enter; it is not within my province this afternoon. There, demonstrably, Germany is in the exercise of an instrument of precision which we do not possess, and which we are never likely to possess in the same degree. What I am thinking of rather when I speak of the State in connection with responsibility is the feeding of the hungry, the clothing of the naked, and the healing of the sick and the afflicted. In our previous anxiety to push these things on to other shoulders than our own we over-reached ourselves. We see now, or we ought to see, that the voluntary principle can never be dispensed with without more loss in the feeding of the hungry, the clothing of the naked, in the education of the young, and, I think I might add, in the provision of due nourishment for the soul.

### Hospitals' Fresh Responsibilities.

We agree that, so far as spiritual effort pure and simple is concerned, the less the State has to do with the matter the better. And the same might be true—at least the tendency has been to make it true—with regard to other things. And yet we are insisting all the time that in the education of the young, parental responsibility should decrease, and that of the State should increase. Let us learn our lesson. Our ordinary methods have proved utterly inadequate, though amid the din of warfare we have not yet paid the attention to the fact that we undoubtedly shall when the war is over. All our needs are as usual, though we are in danger of forgetting that too in the new and unexampled claim that is being made upon our resources, spiritual and material. Our voluntary hospitals, which every year were finding it harder to maintain their ground before the war, have suddenly had their responsibilities quadrupled, their means remaining, broadly, what they were before. They have had to undertake an amount of new work for which they were not designed, more than they ever dreamed they could. They have had to assume a considerable portion of the burden of caring for our wounded soldiers. But the civilian poor still exist; they still go on being ill and dying; they still have to be treated by the institutions which formerly were almost solely appropriated for their benefit. Accommodation has to be found for them, somehow and somewhere, and medical and surgical attendance too, notwithstanding the portentous fact that no small proportion of our hospital staffs, both doctors and nurses, have had to enter directly into the service of the Army. In one voluntary hospital of which I know

* An address given at the Annual Meeting of the League of Mercy at St. James's Palace, December 19.

a little, as I happen to be chaplain of it, we have had an instructive illustration of what this state of things inevitably means. The whole of one floor has had to be given up to the wounded soldiers, accommodation being found for other patients by improvisations of various kinds, even to the using of private houses near by. And yet the number of these civilian patients is not smaller than it was before the war; it is actually greater, the reason being, in part, that so many munition workers suffer from accidents of various kinds, and so many women have now begun to do work which was formerly reserved for men. I will not weary you with details about it: you are all acquainted with the like, more or less. But the moral to be drawn, in the case of that one voluntary hospital, is that there has never been a time of greater and more continued strain upon our voluntary resources, and never a time when, judging by normal standards, they were less able, you would think, to respond to it.

### An Unsurpassed Efficiency.

And yet our voluntary hospitals have responded, as we have been hearing this afternoon, in a most amazing way without cessation and without much public encouragement. Their depleted staffs have worked in season and out of season, day and night, always under pressure more or less, without relaxation of endeavour, and, I think we may proudly claim, with an efficiency unsurpassed, either in war or in peace. And, your Royal Highness, what this ought to teach us is, I think, that it would be a sorry day for our country if ever the voluntary principle was done away with with regard to the care of our sick. No social tendency is irrevocable in any age. We get into the way of thinking so, but it is an entire delusion. After the war we shall begin *de novo* and revise a good many of our commonplaces with regard to this. God grant that the policy of drift and muddle in our public affairs may cease! I am afraid that is too much to hope, because drift and muddle are characteristic of the age, though, somehow, we manage to come through. But may we not also hope that the tendency to eliminate private interest in works of mercy of all kinds may fail? Is it a good thing or is it a bad thing that, so far, your Society and such societies and all private generosity have kept our hospitals from becoming mere departments of the State, ruled by State officials? Is it well that private benevolence should be pulled upon on behalf of our suffering poor? We know the answer. We do not want all heart driven out of our healing energies.

### The Association of Religion and Medicine.

If I had my way—though few of you might be prepared to agree with me—I would like to see the healing art more closely united with the Church. Religion and medicine, in my view, should never have been dissociated as they have been. Whether that is the fault of the Church or of the medical profession I do not know, or whether it is due to public opinion. But I wish the day would come again when they would work together as they did of old, when the only hospitals were religious houses, and when the care of the sick, as of the destitute, was part of the ordinary service of religion. There was no need of any Poor Law, and neither, as a rule, was there any fear of starvation. One would like to get the religious motive back into those who minister to the relief of human suffering and pain. But almost the last shred of that motive is to be seen in the fact that the Churches are expected to contribute to the support of the voluntary hospitals. I hope that will be increased, rather than diminished, as time goes on. One main object of the League of Mercy appears to be the promotion of this very thing—namely, increasing the amount of individual effort and interest in the work of the hospitals. You have insisted on the importance of interesting the many, rather than being dependent on the benefactions of the comparatively few; and now one learns, with much satisfaction, you are extending your operations so as to cover the entire kingdom. This is a momentous departure, and ought to be welcomed everywhere, though it should be recognised at the same time, as of course we do, that London's needs will always be paramount, if only for the reason that London so largely starts the rest of the country.

What impressed me most during my visit to the Front—and I think I saw most of the field—was the high standard of excellence maintained by the hospitals under what would have appeared almost impossible conditions. I am speaking of all the hospitals, and not of the military hospitals alone. And I ought to remark that the military have had to rely largely upon voluntary organisations for their staffing requirements. I think I scarcely ever went into a military hospital without meeting someone, either a doctor or a nurse, whom I had known before in connection with some London hospital. I scarcely went into any hospital where I did not see someone who came from you. And the miracle of that organisation, and the humanity of it, on the testimony of German prisoners themselves, in contrast to the callousness of German methods, and the humane consideration given to the wounded in our hospitals, is striking in the extreme. The German surgeon only seems to take trouble with a wounded man if he can be made fit for the firing-line again—and that is a sign of their State efficiency. Otherwise he may perish; there is no sentimentality wasted upon him. Ours, on the other hand, labour to ease pain and save life, regardless of its value as "cannon fodder."

### Endure to the End.

The letters which reach me from the Front from time to time strike me with one particular note. It is the note of endurance. Whatever may come of the peace proposals, about which we are hearing, I think it ought not to be generally assumed that our men do not know what they are up against. They know perfectly well. There is no pretence that everything is going as well as could be desired. On the contrary, I think there is a grimmer realisation of the stupendous nature of the task we are confronted with, and that it will require our last ounce of strength to win. Is the nation, I wonder, facing the struggle in a similar spirit? Are we prepared to give ourselves, in our degree and at God's call, as heroically and as utterly as are those who are fighting our battles for us? Do we at home yet know the meaning of privation and sacrifice, though we are beginning to? There are thousands of our men and women—and I do not think they are confined to any particular class—who do not yet seem to realise it. How far are we ready to go in acceptance of suffering and willingness to bear one another's burdens, "and so fulfil the law of Christ"? We shall see—and it cannot be long before we see; we cannot afford to wait. And amongst the objects which demand our uttermost devotion, and whose needs are multiplied tenfold by the exigencies of the war, we cannot give the hospitals a second place. Pray God that the greatness of the ministry we are unitedly able to render may be equal to the greatness of the demand.

